# A Preclinical Animal Study of Combined Intragastric Balloon and Duodenal-Jejunal Bypass Liner for Obesity and Metabolic Disease

**DOI:** 10.14309/ctg.0000000000000234

**Published:** 2020-09-21

**Authors:** Hassan Ghoz, Veeravich Jaruvongvanich, Reem Matar, Azizullah Beran, Daniel B. Maselli, Andrew C. Storm, Barham K. Abu Dayyeh

**Affiliations:** 1 Division of Gastroenterology and Hepatology, Mayo Clinic, Rochester Minnesota, USA;; 2 Division of Gastroenterology and Hepatology, Mayo Clinic, Jacksonville, Florida, USA.

## Abstract

**INTRODUCTION:**

Endoscopic bariatric and metabolic therapies can potentially reproduce similar gastric and small intestinal anatomic and physiologic manipulations as Roux-en-Y gastric bypass. This proof of concept animal study was aimed to assess the feasibility, safety, efficacy, and impact on gastrointestinal physiology of combined intragastric balloons (IGB) and duodenal-jejunal bypass liner (DJBL) for the treatment of obesity.

**METHODS:**

Five Ossabaw pigs were fed a high-calorie diet to develop obesity and were randomly assigned to receive IGB or DJBL in sequence. The weight gain rate was calculated. Fasting and postprandial blood samples were drawn before any intervention (serving as the baseline group) and 1 month after second device insertion (serving as the combination group) to measure gut neurohormonal changes and metabolic parameters.

**RESULTS:**

Four pigs successfully received a sequential device insertion. One pig developed duodenal sleeve prolapse that was spontaneously resolved. One pig was early terminated because of developing a central line infection. The rate of weight gain in the combination group (0.63 ± 1.3 kg/wk) was significantly lower than the baseline group (1.96 ± 2.17 kg/wk) and numerically lower than after insertion of the IGB (1.00 ± 1.40 kg/wk) or the DJBL (0.75 ± 2.27 kg/wk) alone. A trend of higher postprandial glucagon-like peptide-1 was observed in the combination group compared with the baseline group.

**DISCUSSION:**

A combination of IGB and DJBL is feasible and well tolerated. A strategy of sequential use of these devices might offer a synergistic approach that can enhance weight loss and metabolic outcomes.

## INTRODUCTION

Roux-en-Y gastric bypass (RYGB) is one of the most effective obesity treatments (1). However, RYGB is invasive, irreversible, with limited acceptance among patients who qualify for it (2–4). With these and other limitations, bariatric surgery has been performed in less than 1%–2% of the eligible candidates in the United States (5), highlighting the need for safer anatomy-preserving bariatric interventions. Endoscopic bariatric and metabolic therapies (EBMTs) have been developed that can potentially reproduce the restrictive and physiologic gastric and small intestinal alterations of bariatric surgery (6). Intragastric balloon (IGB) is a space-occupying device that induces weight loss primarily by a restrictive effect (7). Duodenal-jejunal bypass liner (DJBL) is a 60-cm sleeve that coats the small bowel bypassing its absorptive capabilities and altering its metabolic and gut hormonal response (8,9). Both devices have shown promising results for excess adiposity and metabolic consequences in humans (10,11). IGB improves insulin resistance primarily through weight loss-dependent pathways mediated by alteration in satiety and satiation through its effects on gastric motility and accommodation with no significant gut hormonal alterations (12–15). In some studies, ghrelin decreased during IGB implantation and returned to baseline values after device removal (13,16). DJBL has demonstrated several metabolic benefits that result in complimentary and weight loss-independent improvement in glucose metabolism and insulin resistance through alteration in multiple gut hormones such as peptide YY, glucagon-like peptide-1 (GLP-1), and ghrelin (17).

A combination of these 2 devices may facilitate greater weight loss and impact on obesity-related comorbidities by working through different physiological pathways mimicking RYGB. Our study aimed to assess the feasibility, safety, and explore incremental physiologic and metabolic benefits of the combination in an obese pig model.

## MATERIALS AND METHODS

**Figure 1.**
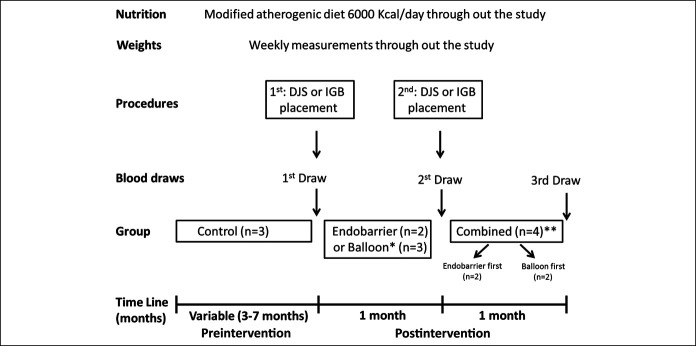
Study outline. DJS, duodenal-jejunal bypass; IGB, Intragastric balloon. *One of the “IGB group” pigs was euthanized after 1 month and did not complete the study (data included). **One of the “combined group” pig only had the combined devices for 2 weeks (data included).

Five Ossabaw miniature pigs aged 5–10 months were enrolled from Indiana University Animal Facility. Each pig received a hypercaloric modified atherogenic diet (6,000 kcal/d, 46% fat) to induce obesity, as previously described (18). The weight induction period served as the baseline group to measure the rate of weight gain per month. All pigs were fed the same measured diet with free access 6 hours daily. Pigs were housed separately, and their daily feds were measured. A tunneled central line catheter was placed in all animals to allow blood draws and measurement of metabolic and gut neurohormonal response to feeding. Pigs were euthanized at the end of the study, as previously described (19). The study flow diagram is depicted in Figure 1. Our study was approved by our Institutional Animal Care and Use Committee (IACUC) and followed the American Association for Laboratory Animal Science guidelines.

### Study protocol

**Figure 2.**
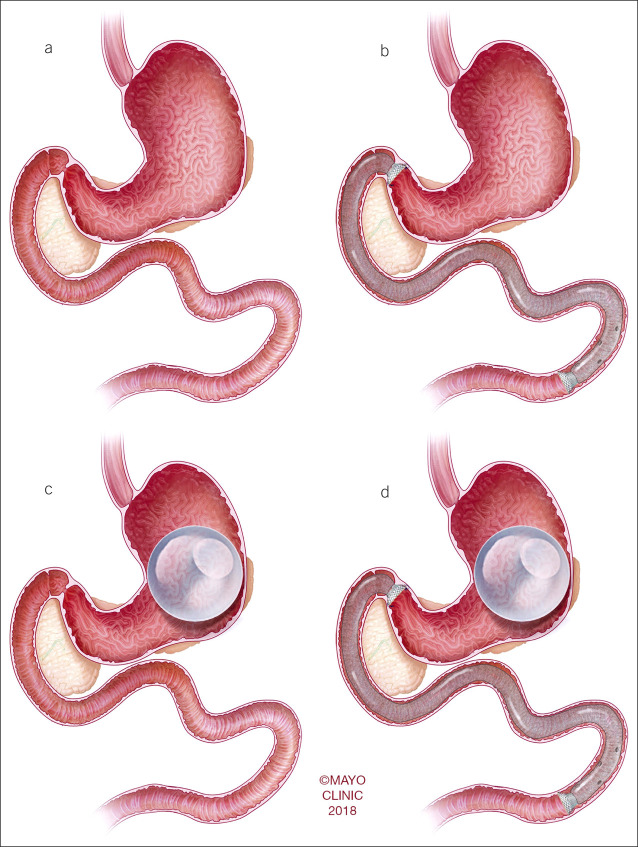
Device placement in (**a**) baseline group, (**b**) duodenal-jejunal bypass sleeve group, (**c**) intragastric balloon group, and (**d**) combination group.

Pigs were randomly assigned to receive IGB (Orbera, Apollo Endosurgery, Austin, TX) or DJBL (Endobarrier, GI Dynamics, Boston, MA) as their first procedure, categorized as the single device group (IGB group or DJBL group). Pigs were observed for 1 month and then received an addition of either IGB or DJBL to their on-going bariatric intervention. The pigs with both IGB and DJBL were categorized as the combination group and were observed for 1 month and then euthanized. Figure 2 outlines the device placement in each group.

### Physical and laboratory measurements

Body weights were measured at the beginning of the study and weekly after that. The primary outcome was the rate of weight gain on a high caloric diet per week. After the pigs were euthanized, a necropsy was performed to examine the location of both devices and evidence of complications.

Of the 5 pigs, 3 pigs completed a 1-month follow-up after sequential device insertion. Fasting and postprandial (15, 45, and 90 minutes) blood samples were obtained at baseline before the first device insertion and 1 month after the second device insertion in these 3 animals.

### Statistical analysis

Data were expressed as mean ± SD. The difference between each group was examined using the Mann-Whitney *U* test. Postprandial metabolic parameter changes were expressed as an area under the curve (AUC). *P*-value of less than 0.05 was considered significant. Data analysis was performed using JMP Pro 14.1 (SAS Institute, Cary, NC).

## Ethical Approval

Our study was in accordance with the ethical animal research guidelines and approved by the American Association for Laboratory Animal Science and the local IACUC board.

## RESULTS

**Figure 3.**
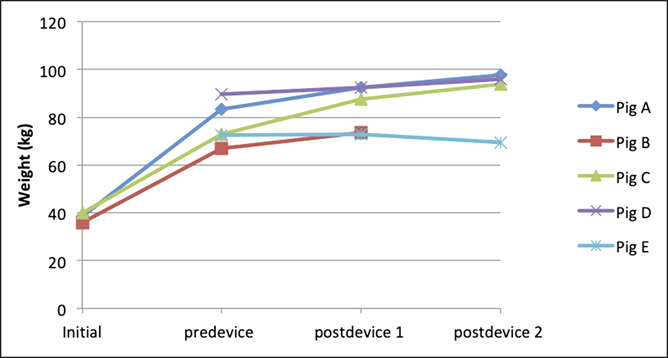
Weight data of each pig. Weight induction period—Pig A (7 months), Pig B (3 months), and Pig C (4 months). Pig B developed septicemia before the second device insertion.

Five pigs were used for this study. Pig A (38.5 kg), Pig B (36.0 kg), and Pig C (40.0 kg) underwent an initial weight induction period of 7, 3, and 4 months, respectively, during which they developed obesity serving as the baseline group. Their weights before device insertion were 83.5, 67, and 73 kg, respectively. Pigs A–C were then randomized to IGB or DJBL. At the time of randomization of Pigs A–C, Pig D (89.5 kg) and Pig E (72.5 kg), who were already obese, were also randomized to IGB or DJBS. Despite the timeline difference of randomization, Pigs D and E were fed the same amount of calories of 6,000 kcal as the other 3 pigs to achieve obesity. Figure 3 details the individual weight data of each pig.

### Feasibility and safety

Four pigs successfully received a sequential device insertion. The fifth pig (Pig B) developed central venous catheter infection 1 month after the IGB placement. The pig was immediately terminated from the study secondary to sepsis. Necropsy did not show any IGB-related adverse events. Three pigs (Pig A, C, and D) had no complications and tolerated the devices well. The fourth pig (Pig E) developed persistent vomiting after 2 weeks of the DJBL insertion. An urgent endoscopy revealed an intestinal obstruction secondary to a sleeve prolapse resolved by liquid and air injection. A following contrast imaging study revealed a spontaneous resolution of obstruction. The weight data in the first 2 weeks after the DJBL insertion before developing the small bowel obstruction of Pig E were used to calculate the weight gain rate in the combination group. A necropsy was performed in all pigs at the study conclusion. All devices were in a good position. There was no evidence of esophagitis, gastric perforation, gastric ulceration, liver abscess formation, pancreatitis, or mesenteric venous thrombosis. Expected superficial ulcerations were seen in the duodenum bulb corresponding to the DJBL anchoring barbs with no high-risk stigmata for bleeding or perforation (see Figures 1 and 2, Supplementary Digital Contents 1 and 2, http://links.lww.com/CTG/A385, http://links.lww.com/CTG/A386).

### Efficacy

**Figure 4.**
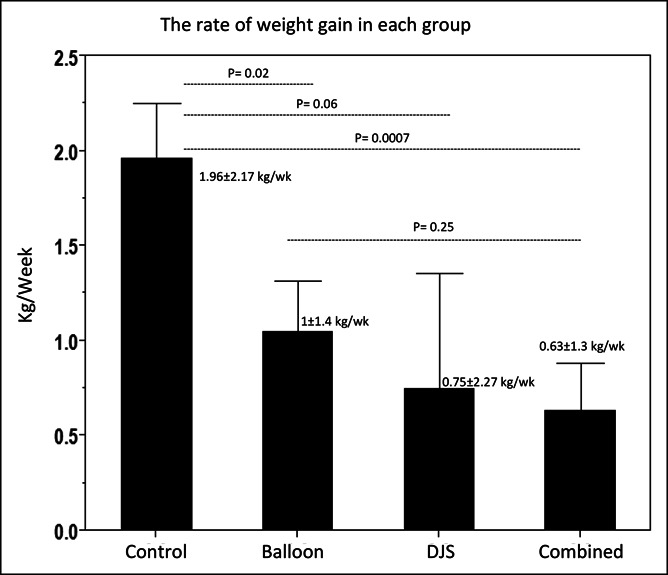
The rate of weight gain of each group. DJS, duodenal-jejunal bypass.

The baseline group (Pig A, B, and C) had a weight gain rate of 1.96 ± 2.17 kg/wk. Weight gain rates were significantly lower in the IGB group (Pig B, C, and E) (1.0 ± 1.4 kg/wk, *P* = 0.02), DJBL group (Pig B and D) (0.75 ± 2.27 kg/wk, *P* = 0.06), and combination group (Pig A, C, D, and E) (0.63 ± 1.3 kg/wk, *P* < 0.001) than the baseline group. The weight gain rate of the combined devices was numerically lower than each device individually (*P* = 0.25) and similar regardless of the sequence of the device insertion (*P* = 0.90) (Figure 4).

### Metabolic and physiologic alteration

**Figure 5.**
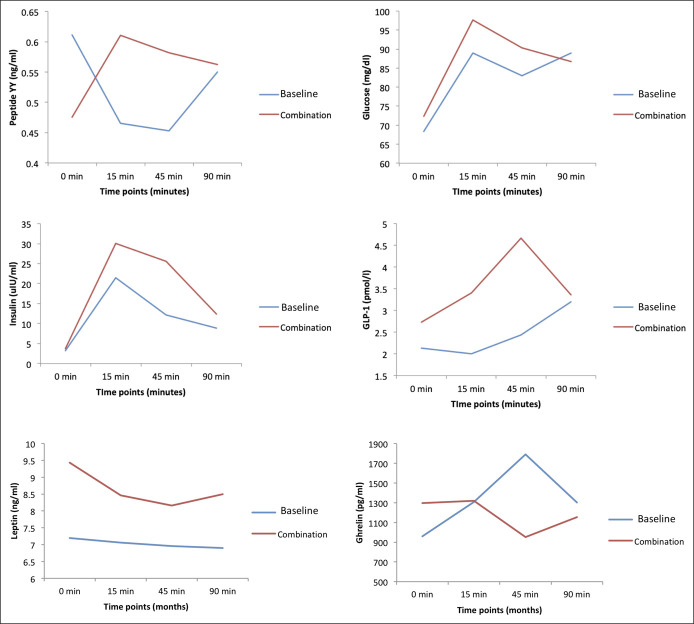
Postprandial metabolic parameters.

Of 3 pigs (Pig A, C, and D) that completed the one-month follow-up after a sequential device insertion, fasting blood samples for the metabolic and gut hormonal profile did not differ significantly in the combination group compared with baseline measurements in the same pigs (Table 1). For postprandial blood samples, there was a trend of higher AUC of GLP-1 and lower AUC of ghrelin in the combination group than the baseline measurements in the same pigs (Table 2 and Figure 5).

**Table 1.**
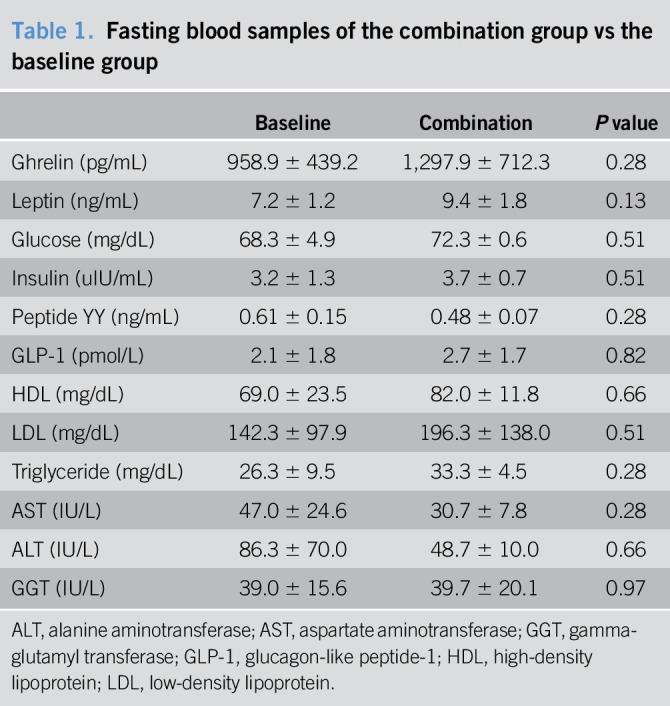
Fasting blood samples of the combination group vs the baseline group

	Baseline	Combination	*P* value
Ghrelin (pg/mL)	958.9 ± 439.2	1,297.9 ± 712.3	0.28
Leptin (ng/mL)	7.2 ± 1.2	9.4 ± 1.8	0.13
Glucose (mg/dL)	68.3 ± 4.9	72.3 ± 0.6	0.51
Insulin (uIU/mL)	3.2 ± 1.3	3.7 ± 0.7	0.51
Peptide YY (ng/mL)	0.61 ± 0.15	0.48 ± 0.07	0.28
GLP-1 (pmol/L)	2.1 ± 1.8	2.7 ± 1.7	0.82
HDL (mg/dL)	69.0 ± 23.5	82.0 ± 11.8	0.66
LDL (mg/dL)	142.3 ± 97.9	196.3 ± 138.0	0.51
Triglyceride (mg/dL)	26.3 ± 9.5	33.3 ± 4.5	0.28
AST (IU/L)	47.0 ± 24.6	30.7 ± 7.8	0.28
ALT (IU/L)	86.3 ± 70.0	48.7 ± 10.0	0.66
GGT (IU/L)	39.0 ± 15.6	39.7 ± 20.1	0.97

ALT, alanine aminotransferase; AST, aspartate aminotransferase; GGT, gamma-glutamyl transferase; GLP-1, glucagon-like peptide-1; HDL, high-density lipoprotein; LDL, low-density lipoprotein.

**Table 2.**
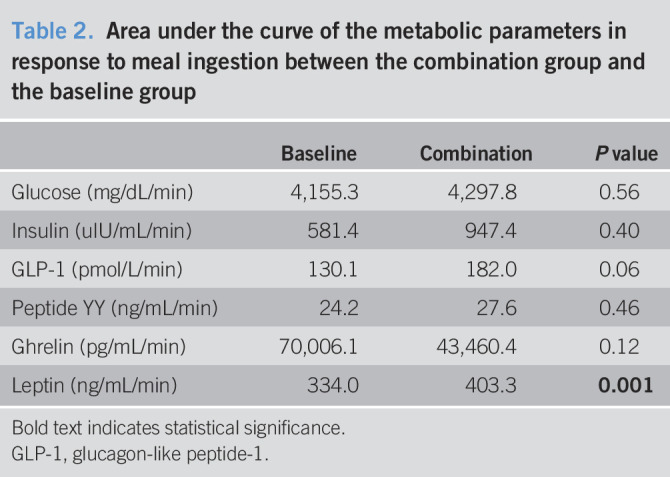
Area under the curve of the metabolic parameters in response to meal ingestion between the combination group and the baseline group

	Baseline	Combination	*P* value
Glucose (mg/dL/min)	4,155.3	4,297.8	0.56
Insulin (uIU/mL/min)	581.4	947.4	0.40
GLP-1 (pmol/L/min)	130.1	182.0	0.06
Peptide YY (ng/mL/min)	24.2	27.6	0.46
Ghrelin (pg/mL/min)	70,006.1	43,460.4	0.12
Leptin (ng/mL/min)	334.0	403.3	**0.001**

Bold text indicates statistical significance.

GLP-1, glucagon-like peptide-1.

## DISCUSSION

Our preclinical animal study of the combined IGB and DJBL treatment demonstrated that this treatment strategy is feasible and potentially effective as 4 pigs successfully received a sequential device insertion. Regarding safety, 3 pigs tolerated the devices well and completed the study, and both devices were in place on necropsy. One pig developed an intestinal obstruction because of a DJBL prolapse that spontaneously resolved. Worldwide registry data of 492 patients with DJBL found that the rate of device migration was 3% with an intestinal obstruction of 0.3% of cases (20,21). Central line infection in one pig in our study was not related to either study device.

We had hypothesized that the combination of IGB and DJBS would enhance weight loss because they work through different mechanistic pathways in the stomach and small intestines (10,11). Our study demonstrated that the weight gain rate of the combination group was significantly lower than the baseline group and numerically lower than each device individually, but this did not reach statistical significance. This could be from a small sample size or a potentially overlapping mechanism of both devices. Delayed gastric emptying is not only a primary weight loss mechanism of IGB (22); this effect was also observed after DJBS (21,23). A previous study of 7 Ossabaw miniature pigs aged 5–10 months receiving the same type of diet reported weight gain of 37.7 ± 11.8 kg in 24 weeks, which is 1.57 kg/wk on average (18). This weight gain rate is numerically comparable with our baseline group and higher than our treatment groups, further corroborating our findings.

We also observed a trend toward higher GLP-1 in the combination group. However, changes in metabolic and gut hormonal profiles reported in our study are exploratory, given the small sample size and limitations of our animal model. This finding alludes to the potential additive weight loss-independent benefit of using DJBS with or after IGB therapy to improve diabetes remission rates and resolution of nonalcoholic fatty liver disease (24–27). Because both gastric and small intestinal EBMTs are available clinically, the implication to clinical practice and the management of obesity and its comorbidities as a chronic disease is clear.

Our design as a feasibility study led to small numbers with low statistical power. However, the supply and cost of Ossabaw mini pigs are prohibitive to conduct larger studies or have a true control group studied in parallel. However, the metabolic and gut hormonal profiles after sequential device insertion were directly compared with their baseline values before device insertion in the same pigs serving as the baseline group. To demonstrate the physiologic viability of our approach, we had to use this pig model. Unlike other porcine models, they have a natural tendency to deposit excess fat and develop obesity-related diseases with its metabolic consequences when fed a high-calorie diet.

In summary, a combination of gastric and small intestinal EBMT is feasible and well-tolerated in a large animal model. A strategy of the sequential use of these devices might enhance weight loss durability and obesity comorbidities resolution. Further studies should be carried out prospectively in human subjects. However, findings from this study will usher a new ear in bariatric and metabolic endoscopy that harness the power of the gastrointestinal tract for the treatment of obesity and its metabolic consequences.

## CONFLICTS OF INTEREST

**Guarantor of the article:** Barham K. Abu Dayyeh, MD, MPH.

**Specific author contributions:** Hassan Ghoz, MB, BCh, and Veeravich Jaruvongvanich, MD, have equal contribution to this work as co-first authors. H.G. and B.K.A.D. conceived and designed of the study; reviewed the literature; collected, analyzed, and interpreted the data; and drafted the manuscript. D.B.M., B.K.A.D., R.M. conceived and designed the study and critically revised the manuscript. V.J., B.K.A.D., and R.M. reviewed the literature; collected, analyzed, and interpreted the data; and drafted the manuscript. All authors read and approved the final manuscript.

**Financial support:** Mayo Clinic Internal funding. Devices were provided by GI Dynamics and Apollo Endosurgery.

**Potential competing interests:** A.C.S. has received research support from Boston Scientific and has served as a consultant for Apollo Endosurgery, GI Dynamics, and Endo-TAGSS. B.K.A.D. has received research support from Apollo Endosurgery, USGI Medical, GI Dynamics, Carin Diagnostics, Medtronic, Boston Scientific, Spatz Medical. He has served as a consultant to Boston Scientific and USGI Medical. He has served as a speaker to Endogastric Solutions, Olympus, and Johnson & Johnson.

**Study Highlights**

**WHAT IS KNOWN**

✓ RYGB is one of the most effective obesity treatments. However, it is invasive, irreversible, with limited acceptance among patients who qualify for it, highlighting the need for safer anatomy-preserving bariatric interventions.

**WHAT IS NEW HERE**

✓ A combination of 2 EBMTs, namely IGB and DJBL, is feasible and well-tolerated in a pig model.

**TRANSLATIONAL IMPACT**

✓ A strategy of sequential use of these devices might offer a synergistic approach that can enhance weight loss and metabolic outcomes.

## Supplementary Material

SUPPLEMENTARY MATERIAL
