# Supplementary figures and images for: A Preclinical Animal Study of Combined Intragastric Balloon and Duodenal-Jejunal Bypass Liner for Obesity and Metabolic Disease
Source: Clin Transl Gastroenterol. 2020 Sep 21;11(9):e00234. doi: 10.14309/ctg.0000000000000234 (PMC7508443; doi:10.14309/ctg.0000000000000234)

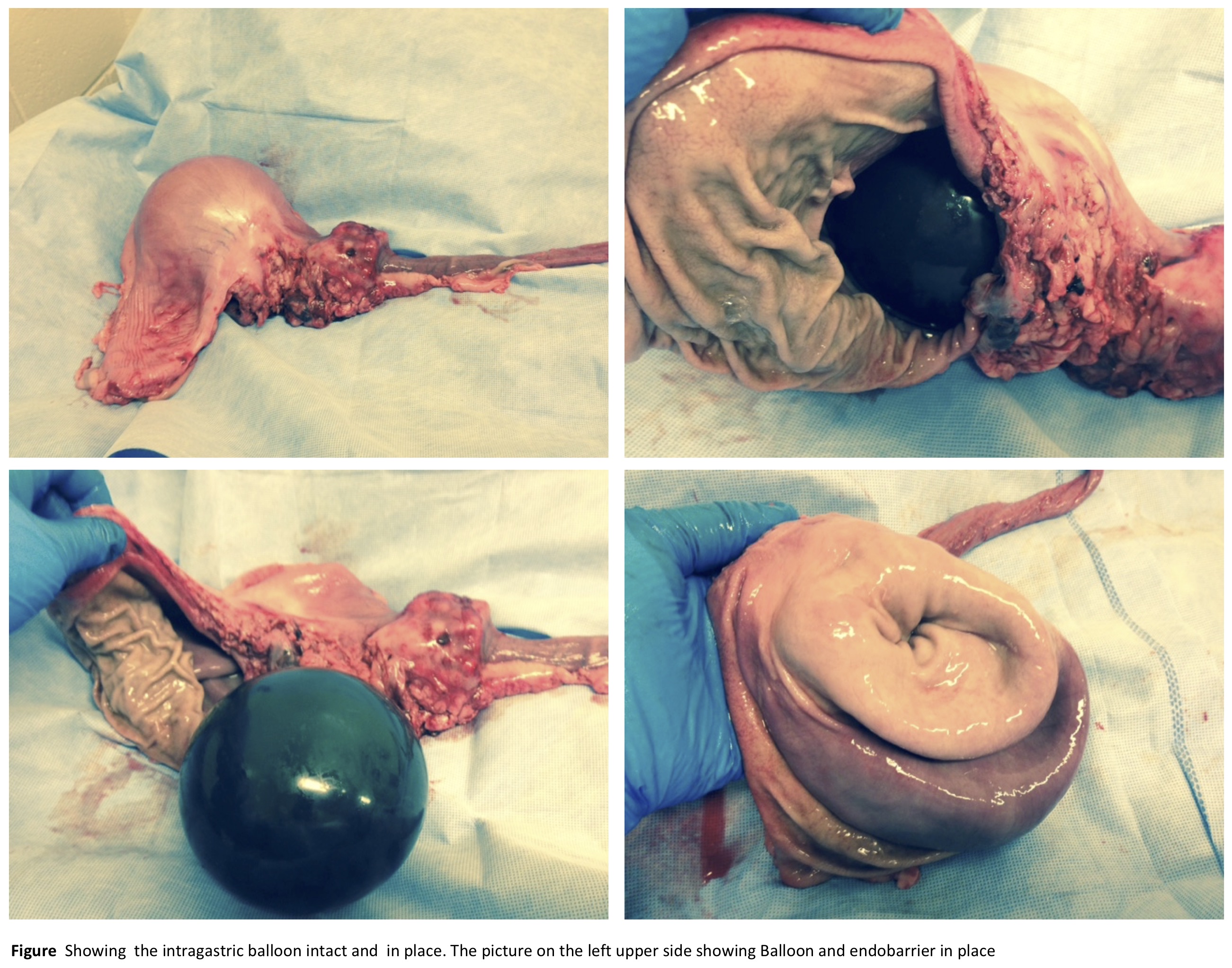

Supplement: SUPPLEMENTARY MATERIAL [file ct9-11-e00234-s001.jpg]

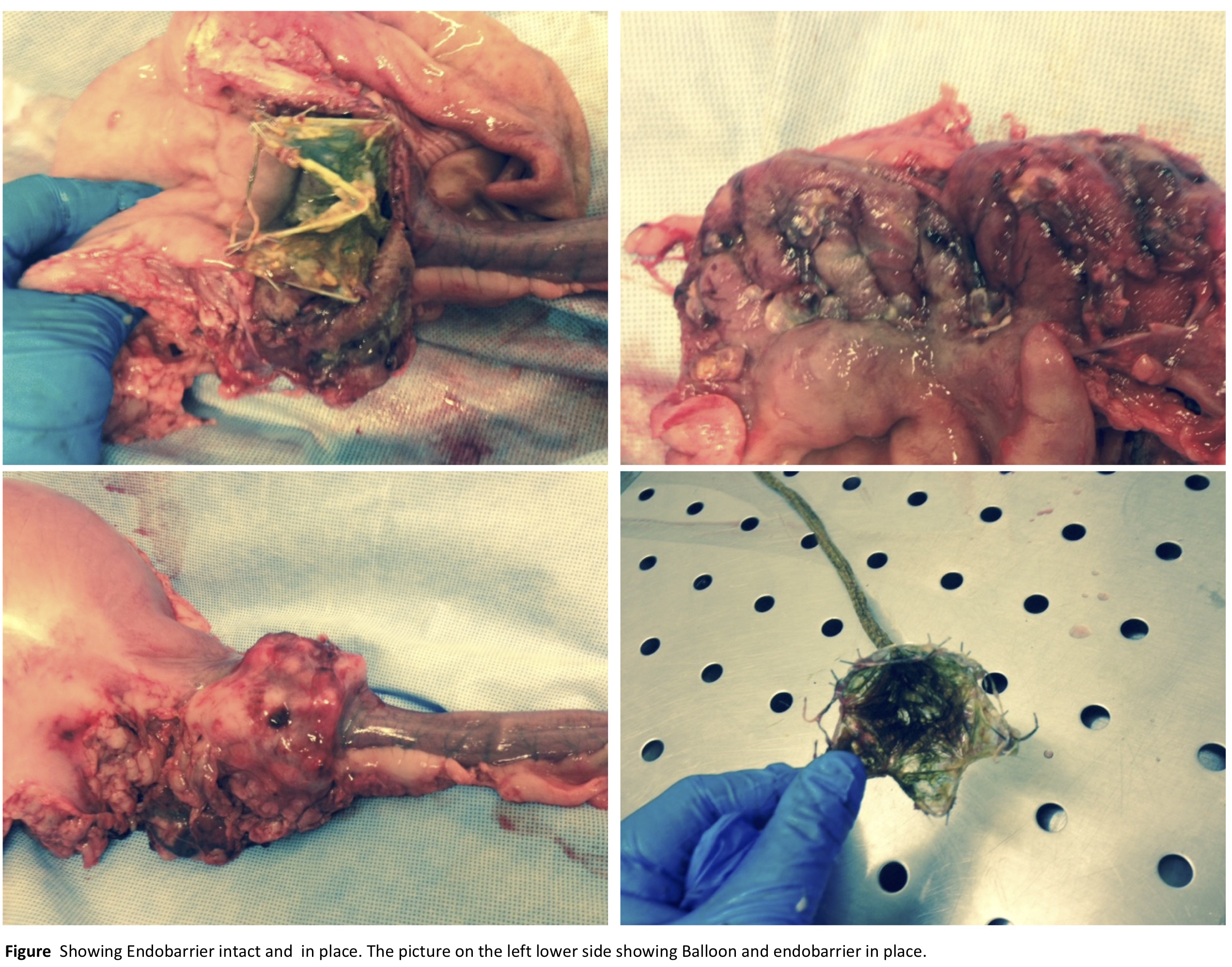

Supplement: SUPPLEMENTARY MATERIAL [file ct9-11-e00234-s002.jpg]
